# ﻿*Petrocodoncurvitubus*, a new species of Gesneriaceae from Guangxi, China

**DOI:** 10.3897/phytokeys.252.136306

**Published:** 2025-02-12

**Authors:** Jin-Xin Wei, Bo Pan, Tao Ding

**Affiliations:** 1 Guangxi Key Laboratory of Plant Conservation and Restoration Ecology in Karst Terrain, Guangxi Institute of Botany, Guangxi Zhuang Autonomous Region and Chinese Academy of Sciences, CN-541006, Guilin, Guangxi Zhuang Autonomous Region, China Guangxi Institute of Botany, Guangxi Zhuang Autonomous Region and Chinese Academy of Sciences Guilin China; 2 Guilin Xing’an Lijiangyuan Forest Ecosystem Observation and Research Station of Guangxi, CN-541316, Guilin, Guangxi Zhuang Autonomous Region, China Guilin Xing’an Lijiangyuan Forest Ecosystem Observation and Research Station of Guangxi Guilin China

**Keywords:** China, Gesneriaceae, Guangxi, limestone flora, new species

## Abstract

A new species of Gesneriaceae, *Petrocodoncurvitubus* J.X.Wei, B.Pan & T.Ding, **sp. nov.** from Guangxi, China, is described and illustrated. The new species is morphologically similar to *P.lui* and *P.tenuitubus*, but can be easily distinguished by its elliptic to oblong-ovate leaves, corollas with two purple longitudinal stripes, and conical ovary.

## ﻿Introduction

The genus *Petrocodon* Hance was established in 1883, initially containing only one species, *Petrocodondealbatus* Hance (Hance, 1883). For over a century, the genus remained monotypic until two new species, *P.ferrugineus* Y.G. Wei and *P.multiflorus* F. Wen & Y.S. Jiang, were discovered in southern China (Wei 2007; Wei et al. 2010). In 2011, the range and morphological diversity of the genus were further expanded based on molecular evidence (Wang et al. 2011; Weber et al. 2011), which led to the incorporation of several monotypic genera, such as *Calcareoboea* C.Y.Wu ex H.W.Li, *Dolicholoma* D. Fang & W.T. Wang, *Paralagarosolen* Y.G. Wei, and *Tengia* Chun, as well as all species of the small genus *Lagarosolen* W.T. Wang, four species of *Didymocarpus* Wall., one species of *Wentsaiboea* D. Fang & D.H. Qin, one species of *Primulina* Hance, and recently all but the type species of *Allocheilos* W.T.Wang (Wang et al. 2011; Weber et al. 2011; Xu et al. 2014; Liu et al. 2024). In recent years additional taxa have been discovered in *Petrocodon* such as *P.villosus* Xin Hong, F.Wen & S.B.Zhou, *P.rubrostriatus* K. Tan, X.Q. Song & M.X. Ren, *P.jiangxiensis* F. Wen, L.F. Fu & L.Y. Su, *P.pulchriflorus* Y.B.Lu & Q.Zhang (Hong et al. 2014; Lu et al. 2017; Su et al. 2019; Tan et al. 2023). According to the data from the Gesneriaceae Resource Centre (GRC 2024), there are currently 55 species and one variety of *Petrocodon* (Petrocodondealbatusvar.denticulatus (W.T. Wang) W.T. Wang) recognized worldwide. These species are mainly distributed in southwestern China, on the Indochina Peninsula, northern Thailand, northern Laos, and northern Vietnam. Notably, *P.flavus* D.J. Middleton & Sangvir. is endemic to Thailand; *P.leveilleus* (Fedde) X.X. Bai & F. Wen and *P.hispidus* (W.T.Wang) A. Weber & Mich. Möller are distributed in China and Vietnam; *P.bonii* (Pellegr.) Mich. Möller & A. Weber is distributed in northeastern Thailand and Vietnam; and the remaining *Petrocodon* species are endemic to China (Pellegrin 1926; Li 1982; Vu 2006; Weber et al. 2011; Middleton et al. 2015; Vu 2017; Xin et al. 2021; Xiong et al. 2024).

In January 2024, the authors collected an unidentified species of Gesneriaceae with a purple corolla growing on a stone wall at the entrance of a karst cave in Debao County, Guangxi. This species had a slender corolla, and its curved corolla tube showed a certain similarity to those of *Primulinacurvituba* B. Pan, Li H. Yang & M. Kang (Yang et al. 2017). However, further detailed observation showed that the stigma of the newly collected species was bilobed with oval lobes, unlike the chiritoid stigma of *Primulina* Hance. Other morphological characteristics, such as alternate leaves in basal rosettes, bilobed corolla with two fertile stamens, and capsules dehiscing into four valves, indicate that the species belongs to *Petrocodon* (Weber et al. 2011, 2020). We collected two flowering individuals as voucher specimens, carefully observed plants in the wild, and the herbarium specimens in the laboratory. We also compared them with the digital specimens of the Chinese Virtual Herbarium (https://www.cvh.ac.cn/), JSTOR Global Plants (http://plants.jstor.org), and the latest publication on *Petrocodon* taxa. This species can be clearly distinguished from other accepted *Petrocodon* species. The curved shape of the corolla tube is similar to *P.lui* (Yan Liu & W.B. Xu) A. Weber & Mich. Möller and *P.tenuitubus* W.H. Chen, F. Wen & Y.M. Shui (Xu et al. 2010; Chen et al. 2019), but it can be distinguished from *P.lui* by leaf shape, corolla coloration, bract, corolla tube, stamen, and pistil size. It is different from *P.tenuitubus* in leaf shape and color, number of inflorescences and bracts, corolla shape and indumentum, and pistil shape. Therefore, we describe it as a new species of *Petrocodon*, *P.curvitubus*.

## ﻿Materials and methods

We collected two specimens from the type locality and deposited them at IBK. The morphological measurements and descriptions of the new species are based on plants observed in the field. Additionally, we compared the new species with digital specimens from the Chinese Virtual Herbarium and JSTOR Global Plants and checked the relevant literature (e.g., Xu et al. 2010; Chen et al. 2019). The morphological data for *P.lui* and *P.tenuitubus* used in comparison came from the protologues (Xu et al. 2010; Chen et al. 2019) and their specimens. We also thoroughly examined the type specimen of *P.tenuitubus* (isotype: IBK [IBK00417055]), the type of *P.lui* (holotype: IBK [IBK00406224], isotype: IBK [IBK00406225], paratype: IBK [IBK00406226], [IBK00406227], [IBK00406228], [IBK00406229]). Furthermore, the endangered category of this new species was assessed according to the criteria of the IUCN Red List (IUCN 2024). This new species is described using the terminology used by Wang et al. (1998) and Harris and Harris (2001).

## ﻿Taxonomic treatment

### ﻿ Petrocodon curvitubus

Taxon classificationPlantaeLamialesGesneriaceae

J.X.Wei, B.Pan & T.Ding sp. nov.

CEED921C-54A9-521D-A6AB-8C7DB855CE4A

urn:lsid:ipni.org:names:77356664-1

Figs 1–3

#### Diagnosis.

*P.curvitubus* is florally similar to *P.lui* and *P.tenuitubus*. However, it can be distinguished from these by leaf characteristics, with leaves elliptic to oblong-ovate, apex obtuse or slightly acuminate, base cuneate (vs *P.lui*, ovate or broadly ovate; apex subacute or obtuse, base cordate to shallowly cordate, oblique; vs *P.tenuitubus*, ovate to orbicular; apex obtuse, base cordate symmetrical or asymmetrical), corollas with purple stripes (vs absent in *P.lui* and *P.tenuitubus*), corolla lobes obovate-elliptic to oblong, apex acuminate (vs *P.lui*, lobes oblong, obovate to suborbicular, apex obtuse; vs *P.tenuitubus*, lobes narrowly ovate to ovate, apex obtuse), and ovary conical, ca. 2.5 × ca. 0.8 mm (vs *P.lui*, linear, ca. 3 × 1.5 mm; vs *P.tenuitubus*, ovoid, 1–2 × 1–1.5 mm) (Table 1).

**Table 1.** Comparison of characters among *P.curvitubus*, *P.lui*, and *P.tenuitubus*.

Character	*P.curvitubus* sp. nov.	*P.lui*	*P.tenuitubus*
Leaf	elliptic to oblong-ovate; apex obtuse or slightly acuminate, base cuneate	ovate or broadly ovate; apex subacute or obtuse, base cordate to shallowly cordate, oblique	ovate to orbicular; apex obtuse, base cordate symmetrically or asymmetrically
Leaf adaxially	densely white pubescent	glabrous or sparsely puberulent	densely strigose
Leaf abaxially	densely white pubescent	puberulent	tomentose
Peduncles	densely white pubescent	puberulent	glandular hairs
Bracts	2, adaxially densely white pubescent	2, adaxially puberulent	3, adaxially tomentose
Pedicels	densely white pubescent	puberulent	densely glandular hairs
Corolla tube	slenderly tubular, strongly curved near base, 1–1.3 cm long, 1.5–2.5 mm in diameter at the mouth, with two dark purple longitudinal stripes, stripes glabrous	slender, curved, 1.0–1.5 cm long, ca. 5–6 mm in diameter at the mouth, with two yellow stripes, stripes densely covered with glandular hairs	slender, curved, 0.7–1.6 cm long, 6–8 mm in diameter at mouth, with two yellow stripes, stripes glabrous
Corolla	lobes obovate-elliptic to oblong, apex acuminate	lobes oblong, obovate to suborbicular, apex obtuse	lobes narrowly ovate to ovate, apex obtuse
Filaments	glabrous	sparsely glandular	glabrous
Ovary	conical, ca. 2.5 × ca. 0.8 mm	linear, ca. 3 × 1.5 mm	ovoid, 1–2 × 1–1.5 mm

**Figure 1.**
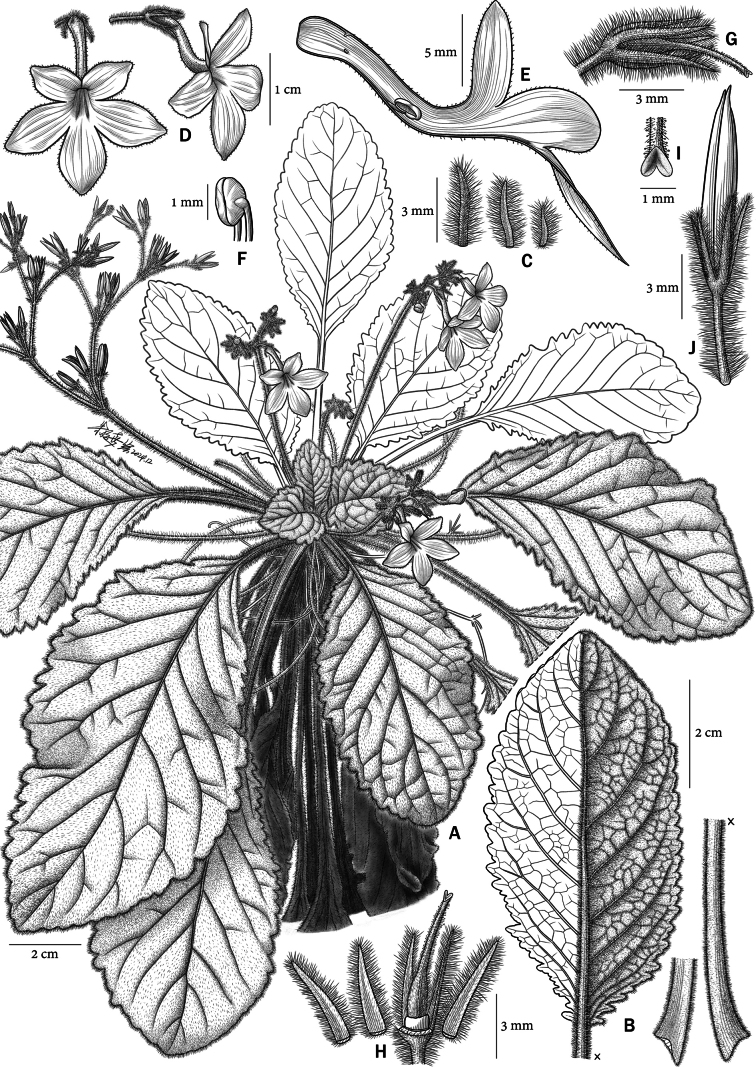
*Petrocodoncurvitubus* J.X.Wei, B.Pan & T.Ding, sp. nov. **A** plant in flower **B** abaxial leaf surface and petiole **C** bracts **D** frontal view of corolla and side view of flower showing strongly curved corolla tube **E** longitudinal section of corolla showing the position of stamens **F** stamens with cohering anthers **G** pistil and calyx **H** pistil with sepals dissected **I** stigmas **J** fruit.

#### Description.

Perennial herbs. Rhizome short and straight, subterete, 1–3.8 cm long, 7.5–9 mm in diameter. Leaves 10–18, basal, alternate, and congested at rhizome apex; petiole terete, adaxially pale green, abaxially pale brown, 2.5–6.4 cm long, 2–2.5 mm in diameter, densely white pubescent. Leaf blade herbaceous, elliptic to oblong-ovate, 2.1–13.3 × 1.1–7.5 cm, apex obtuse or slightly acuminate, base cuneate, margin crenulate, adaxially surface pale green, abaxially grayish green, both surfaces densely white pubescent, lateral veins pinnate 5–8 pairs on each side, adaxially concave, abaxially prominent. Inflorescences 3–6, axillary, 1–5-branched, cymose, (4-)10–16(-22)-flowered; peduncles 2.2–6.8 cm long, 2–2.5 mm in diameter, densely white pubescent; bracts 2, linear, opposite, 4–5 × ca. 0.5 mm, outside densely white pubescent, inside glabrous; pedicels 3–8 mm long, 0.3–0.7 mm in diameter, densely white pubescent. Calyx 5-lobed from base, pale green, lobes linear to triangular, 4–6 × ca. 0.5 mm, outside densely white pubescent, inside glabrous; Corolla pale purple, zygomorphic, 1.8–2.2 cm long, both surfaces sparsely puberulent, with two dark purple longitudinal stripes in the corolla mouth; corolla tube slenderly tubular, strongly curved downwards at base (1–3 mm from the base), then bent forwards, 1–1.3 cm long, 1.5–2.5 mm in diameter at the mouth, 1 mm in diameter at the base, adaxial lip 2-lobed to near base, obovate-elliptic, 0.5–0.7 cm long, ca. 0.3 mm in diameter, apex acuminate; abaxial lip 3-lobed to base, oblong, 0.6–1.1 cm long, 2.5–4 mm in diameter, apex acuminate. Stamens 2, adnate about 6 mm from corolla base, glabrous; anthers pale yellow, ca. 1.5 mm long, dorsifixed, cohering apically; filaments ca. 1 mm long, white, glabrous. Staminodes 3, ca. 0.1 long, white, adnate 2.5 mm above corolla base. Disc annular, ca. 0.5 mm high, orange-yellow. Pistil ca. 5.5 mm long, ovary conical, ca. 2.5 mm long, ca. 0.8 mm in diameter, style ca. 3 mm long, ovary and style densely covered with short glandular and eglandular hairs, stigma bilobed, lobes oval, ca. 0.5 mm long. Capsule 0.5–1 cm long, about 2 mm wide, dehiscing 4-valved. Seeds not seen.

**Figure 2.**
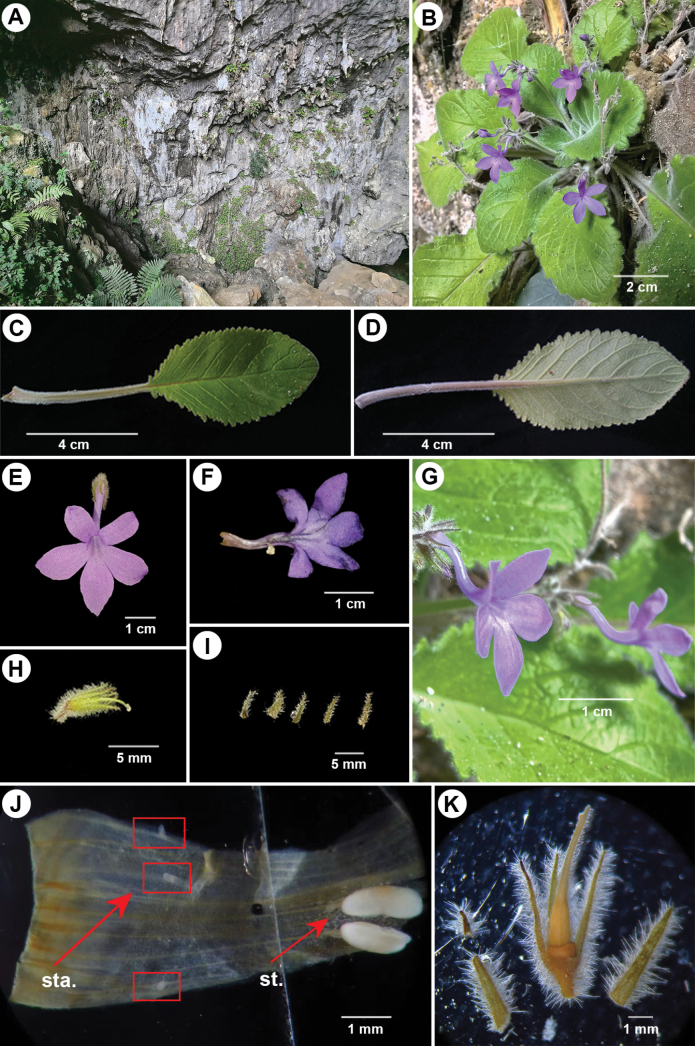
*Petrocodoncurvitubus* J.X.Wei, B.Pan & T.Ding, sp. nov. **A** habitat **B** plant in flower **C** adaxial leaf surface **D** abaxial leaf surface **E** frontal view of flower **F** opened corolla **G** side view of flower showing strongly curved corolla tube **H** pistil and calyx **I** sepals **J** stamens (st.) and staminodes (sta.) from a pickled specimen **K** pistil and calyx with partly dissected sepals from a pickled specimen.

#### Type.

China • Guangxi Zhuang Autonomous Region, Baise City, Debao County, Longguang Town, 23°5'N, 106°44'E, 454 m a.s.l., growing on rock wall at the entrance of karst cave. 26 January 2024, flowering, *WJX001* (holotype: IBK! IBK00470322), *WJX002* (paratype: IBK! IBK00470323).

**Figure 3.**
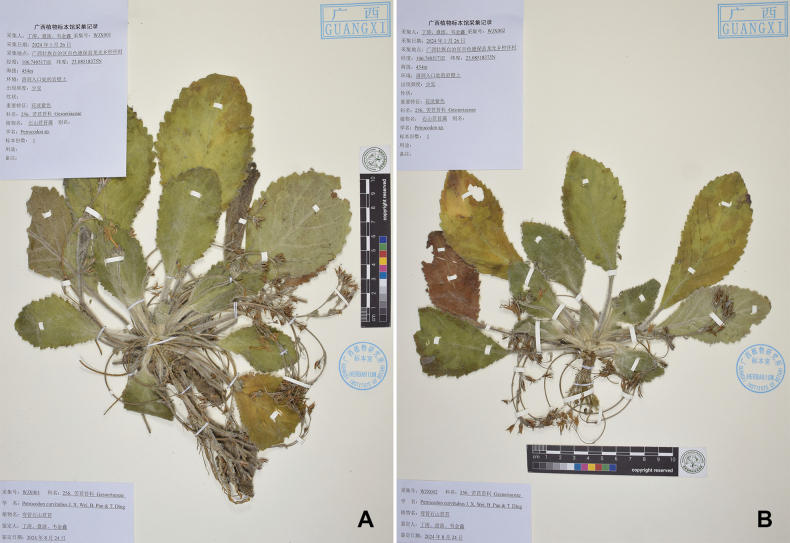
Herbarium type specimens of *P.curvitubus* J.X.Wei, B.Pan & T.Ding, sp. nov. **A** WJX001 **B** WJX002.

#### Phenology.

Flowering from January to March.

#### Etymology.

The epithet originates from the strongly curved corolla tube.

#### Vernacular name.

Wān Guǎn Shí Shān Jù Tái (弯管石山苣苔), the first two words, “Wān Guǎn,” mean the corolla tube is strongly curved, and the following four words, “Shí Shān Jù Tái,” mean *Petrocodon* in Chinese.

#### Distribution and habitat.

*Petrocodoncurvitubus* can only be found at its type locality, Longguang Township, Debao, Baise, Guangxi. It grows on a limestone wall at the entrance of a karst cave at an altitude of 454 m. The average annual temperature in Debao County is 19.5 °C, and the average annual precipitation is 1456.2 mm.

#### Preliminary conservation assessment.

Currently, only one population that consists of about 200 mature individuals of *P.curvitubus* has been discovered. This population is concentrated on the stone wall at the entrance of a karst cave that is located in Longguang Township, Debao, Baise, Guangxi. The area occupied (AOO) is about 100 m^2^, which is significantly smaller than the smallest AOO unit of the IUCN (2024) (10 km^2^ for Critically Endangered under B2). Additionally, we also observed that the habitat of *P.curvitubus* is located near a village and is vulnerable to human activities. Frequent human impacts may lead to a reduction in mature individuals of this species and the decline of the population. According to the IUCN Red List Categories and Criteria (IUCN 2024), the endangered level of this new species is preliminarily assessed as “Critically Endangered” [CR, B2a,b (iii,v)].

## ﻿Discussion

A curved corolla tube can be found in Chinese Gesneriaceae not only in *Petrocodon*, but also in *Primulina* and *Oreocharis* Benth, as exemplified by *P.curvituba*, *Oreochariscurvituba* J.J. Wei & W.B. Xu (Wei et al. 2016) and *Oreocharispumila* (W.T. Wang) Mich. Möller & A. Weber (Möller et al. 2011). It has been hypothesized that a curved corolla tube may represent a pollinator shift and an adaptation to a long-tongued pollinator of these plants (Guo and Wang 2014; Yang et al. 2017). Research on genera such as *Aquilegia* L. and *Oreocharis* has shown that pollinator shifts often drive the evolution of floral traits (Huang 2007; Whittall and Hodges 2007; Jin et al. 2021). However, whether the curved corolla tube of *P.curvitubus* is specifically adapted to certain pollinators requires further investigation. Future studies could include the construction of phylogenetic relationships and floral trait analyses among Gesneriaceae species with curved corolla tubes to better understand their adaptation and evolutionary trajectories.

## Supplementary Material

XML Treatment for Petrocodon curvitubus

## ﻿Additional information

### Author ORCIDs

Jin-Xin Wei https://orcid.org/0009-0004-8879-1544

Bo Pan https://orcid.org/0000-0003-0996-524X

Tao Ding https://orcid.org/0009-0005-7566-8448

### Data availability

All of the data that support the findings of this study are available in the main text.
